# Assessment of Right Ventricular Pressure in Chronic Thromboembolic Pulmonary Hypertension: Comparison of Diagnostic Modalities and Balloon Pulmonary Angioplasty Outcomes

**DOI:** 10.3390/diagnostics15162050

**Published:** 2025-08-15

**Authors:** Gábor Kolodzey, Andrea Péter, Andrea Daragó, László Balogh, Zsuzsanna Bereczky, Judit Barta, Zoltán Csanádi, Tibor Szűk

**Affiliations:** 1 Department of Cardiology, Faculty of Medicine, University of Debrecen, 4032 Debrecen, Hungary; peter.andrea@med.unideb.hu (A.P.); adarago@med.unideb.hu (A.D.); balogh.laszlo@med.unideb.hu (L.B.); barta.judit@med.unideb.hu (J.B.); csanadi.zoltan@med.unideb.hu (Z.C.); 2 Division of Clinical Laboratory Sciences, Department of Laboratory Medicine, Faculty of Medicine, University of Debrecen, 4032 Debrecen, Hungary; zsbereczky@med.unideb.hu

**Keywords:** CTEPH, right ventricular pressure, echocardiography, Swan-Ganz catheterization, pulmonary angiography, BPA

## Abstract

**Background/Objectives:**: Right ventricular (RV) pressure assessment is crucial in both the diagnosis and follow-up of patients with chronic thromboembolic pulmonary hypertension (CTEPH). While right heart catheterization (RHC) and pulmonary angiography (PA) are gold-standard invasive methods, transthoracic echocardiography (TTE) offers a safer and more accessible alternative. This study aimed to evaluate the reliability of echocardiographic RV pressure estimation compared to invasive techniques and to identify clinical predictors of response to balloon pulmonary angioplasty (BPA). **Methods:** In this prospective study, 17 patients with confirmed CTEPH underwent RV pressure assessment via TTE, RHC (Swan-Ganz catheterization), and PA within the same hospitalization period. BPA responders were defined based on clinical improvement and were compared to poor responders using pre- and post-treatment parameters. **Results:** A strong correlation was found between Swan-Ganz and PA-derived pressures (*r* = 0.96), with a slightly lower correlation between TTE and PA (*r* = 0.84), and the lowest between TTE and Swan-Ganz (*r* = 0.78). In the well-responding group, the 6 min walk distance (6MWD) increased by 60 ± 18 m, compared to 12 ± 10 m in poor responders (*p* < 0.01). NT-proBNP levels decreased by 40% in responders versus 10% in non-responders (*p* < 0.01). TAPSE improved significantly in responders (from 16.0 ± 2.0 mm to 19.5 ± 2.5 mm, *p* < 0.01), while RV basal diameter decreased by 15% (*p* < 0.05). No significant echocardiographic improvement was observed in poor responders. **Conclusions:** TTE provides a reliable estimate of RV pressure in CTEPH when standardized protocols are followed. NT-proBNP levels and RV size may serve as useful predictors of BPA response.

## 1. Introduction

Pulmonary embolism is a complex, multi-factorial venous thromboembolic disease [1,2,3], which in 2–4% leads to chronic thromboembolic pulmonary hypertension (CTEPH). CTEPH is caused by persistent organized thromboembolic obstruction of the pulmonary arteries from incomplete resolution of pulmonary embolism [4]. Vascular remodeling contributes to the progress of the disease [5,6,7] with microvasculopathy, increased pulmonary vascular resistance, and progressive pulmonary hypertension [8,9,10]. Establishing the diagnosis of CTEPH remains challenging, as symptoms are often non-specific and typically exacerbated by exertion. Signs of right heart failure generally appear in the advanced stages of the disease. The reported diagnostic delay, extending up to two years [11], is associated with increased morbidity and mortality [12]. Given the progressive nature of the disease, early detection is of paramount importance. Measurement of right ventricular (RV) pressure is a key component in the hemodynamic assessment of patients with CTEPH. It plays a crucial role in diagnostic evaluation, risk stratification and prognostication, treatment planning, as well as patient follow-up [13].

Elevated RV pressure reflects increased pulmonary vascular resistance and provides important insight into disease severity and the functional status of the right heart. Echocardiography is recommended as the first-line, non-invasive diagnostic investigation in suspected pulmonary hypertension [14,15,16] and provides a repeatable and cost-effective means of estimating RV pressure; however, its accuracy in certain clinical scenarios remains a topic of ongoing investigation [17,18]. On the other hand, invasive techniques, such as right heart catheterization and selective pulmonary angiography, offer high accuracy but are associated with higher procedural risks. Recent studies have demonstrated a strong correlation between echocardiographic RV pressure estimates and invasive measurements when standardized imaging protocols are followed [19,20]. Nonetheless, discrepancies can occur due to factors such as suboptimal acoustic windows, arrhythmias, or variations in right ventricular geometry [21]. Given that frequent monitoring of RV pressure is critical in CTEPH management, it is imperative to understand the strengths and limitations of each measurement modality [15].

A multimodal treatment strategy, integrating surgical, interventional, and pharmacological approaches, is increasingly recognized as the optimal management pathway for CTEPH. The primary therapeutic goal in CTEPH is the removal or reperfusion of obstructed pulmonary arteries to reduce pulmonary vascular resistance and unload the right ventricle. The gold standard therapy for centrally located obstructive disease is pulmonary endarterectomy (PEA), which involves the surgical removal of organized thrombi from the proximal pulmonary arteries. PEA yields substantial symptomatic relief, significant hemodynamic improvement, and reduced mortality [22,23,24]. However, up to 40% of patients with CTEPH are not candidates for PEA due to distal, surgically inaccessible disease, unfavorable risk-benefit profiles, or patient refusal. For these patients, alternative treatment modalities have emerged in recent years such as balloon pulmonary angioplasty (BPA) [25,26]. It has now become an integral component of the CTEPH treatment algorithm [15]. Clinical studies from European centers have demonstrated significant improvements in pulmonary hemodynamics—including reductions in mean pulmonary artery pressure (mPAP) by 18–30% and marked decreases in pulmonary vascular resistance (PVR)—alongside improved exercise capacity [27,28]. Anticoagulation is the cornerstone of medical management in all patients with CTEPH, regardless of operability. Lifelong oral anticoagulation is strongly recommended to prevent further thromboembolic events and to stabilize the existing vascular lesions. Vitamin K antagonists (VKAs), such as warfarin, are the standard of care. Riociguat, a soluble guanylate cyclase (sGC) stimulator, is currently the only drug approved specifically for the treatment of inoperable or persistent/recurrent CTEPH after surgery.

In Hungary, BPA was first implemented in Debrecen. The majority of our patients (approx. 70%) consistently accept this treatment approach following appropriate evaluation and shared decision making within the multidisciplinary CTEPH team. Approximately 10% of patients are deemed ineligible for BPA, and an additional 15–20% refuse to undergo the procedure.

This study aims to compare RV pressure measurements obtained by non-invasive echocardiography with those derived from invasive techniques, including right heart catheterization and direct measurement during pulmonary angiography, in a cohort of CTEPH patients. We seek to determine whether echocardiography can reliably substitute for invasive monitoring in routine follow-up and guide therapeutic decision making. Moreover, we intended to explore the potential impact of technical and patient-specific factors on measurement discrepancies, with the goal of improving the accuracy and clinical utility of RV pressure assessments in this high-risk population.

## 2. Materials and Methods

### 2.1. Study Design and Population

An open-label, non-randomized, prospective observational study was conducted at the Department of Cardiology, University of Debrecen. A total of 17 adult patients diagnosed with CTEPH between 2022 and 2025 were included in the study group. The cohort includes both male and female patients across a wide age spectrum. Patients were enrolled following informed consent in accordance with the approved study protocol. All patients were thoroughly informed about the investigational nature of this study, including potential risks and benefits, and gave written informed consent. A prospective analysis was performed to evaluate the correlation between invasive and non-invasive methods used for RV pressure assessment. Measurements were conducted on CTEPH patients within a 48 h window during the same hospital stay to minimize physiological variability. Patients were evaluated for BPA based on clinical parameters and anatomical suitability, as determined by the multidisciplinary CTEPH team.

All relevant demographic and clinical data were collected from hospital records, including vital status, hospitalizations, comorbidities, treatment history, and follow-up outcomes.

### 2.2. Invasive Measurement of the Right Ventricular Pressure

#### 2.2.1. Right Heart Catheterization

Right heart catheterization using a Swan-Ganz catheter is the gold standard method for assessing hemodynamics in patients with suspected or confirmed pulmonary hypertension. The procedure involves the insertion of a balloon-tipped, flow-directed catheter via a central venous access (typically the internal jugular or femoral vein) into the right atrium, right ventricle, and pulmonary artery. It enables direct measurement of pressures within the right heart chambers and pulmonary artery, including right atrial pressure (RAP), right ventricular pressure (RVP), pulmonary artery pressure (PAP), and pulmonary capillary wedge pressure (PCWP). Cardiac output (CO) can also be assessed via thermodilution. Measurements were carried out in supine position using local anesthesia, using standardized equipment to ensure accurate hemodynamic monitoring. For central venous access and thermodilution measurements, an Edwards Lifesciences 831F75 Thermodilution Venous Infusion Port (VIP) catheter was utilized. Vascular access and catheter placement were facilitated by the Intradyn 8F Basic Kit for Intensive Care, which provided all necessary components for safe and sterile insertion. To deliver the injectate required for thermodilution cardiac output assessment, we employed the Edwards CO-SET+ Closed Injectate Delivery System, using room temperature fluid in accordance with established protocols. This closed system ensures reliable and consistent injectate administration while minimizing contamination risks.

#### 2.2.2. Pulmonary Angiography with Invasive RV Pressure Measurement

Diagnostic catheter was advanced through the venous system into the right heart and pulmonary artery. Access site was the femoral vein. Contrast agent was injected to obtain detailed imaging of the pulmonary arteries, identifying obstructions or abnormalities consistent with CTEPH. GE INOVA IG520 system was used. Concurrently, direct measurement of right ventricular pressure was performed.

### 2.3. Non-Invasive Measurement of the Right Ventricular Pressure by Echocardiography

Transthoracic echocardiography was performed using a Philips Epiq 5 Ultrasound System and a phased-array transducer with patients in the left lateral decubitus position. Systolic pulmonary artery pressure (sPAP) was estimated by calculating the pressure gradient across the tricuspid valve using the simplified Bernoulli equation (ΔP = 4 × [TRV]^^2^). The right atrial pressure (RAP) was estimated based on the diameter and inspiratory collapse of the inferior vena cava (IVC), in line with current echocardiographic guidelines. The estimated RAP was then added to the TR gradient to calculate the sPAP as follows:

sPAP = 4 × (TRV)^^2^ + RAP

Measurements were taken from multiple acoustic windows (parasternal, apical, subcostal) to optimize signal quality. The highest quality TR jet envelope was used for analysis. Three consecutive cardiac cycles were averaged for each parameter.

RV function, TAPSE, RV dimensions (basal and mid-cavity diameters), and right atrial area were also assessed. Estimations were based on tricuspid regurgitation velocity (TRV) using continuous-wave Doppler, combined with inferior vena cava (IVC) diameter and its respiratory variability. All studies were performed and interpreted by experienced echocardiographers blinded to the results of invasive measurements.

### 2.4. Balloon Pulmonary Angioplasty (BPA)

BPA was performed as a staged percutaneous intervention in patients found suitable, with a limited number of pulmonary arterial segments targeted during each session. Procedures were guided by selective pulmonary angiography and conducted by an experienced interventional team following institutional protocols.

In a subset of patients undergoing BPA, RV pressure was also recorded invasively during the angiographic procedure, providing an additional possibility for direct comparison of the results of the different methods.

### 2.5. Clinical Assessment

Standardized clinical assessment was performed at baseline for every patient, for patients undergoing BPA, prior to the first BPA, and at three and six months following the final BPA session. Assessments included NYHA functional classification, 6 min walk test (6MWT) distance, NT-proBNP level and the results of the right heart catheterization, pulmonary angiography as well as echocardiography examinations. All laboratory analyses were conducted at the Department of Laboratory Medicine, University of Debrecen, by using standardized protocols and validated assays.

### 2.6. Statistical Analysis

Continuous variables are presented as mean ± standard deviation (SD) or median and interquartile range (IQR), depending on the distribution. Categorical variables are expressed as counts and percentages. The Kolmogorov–Smirnov test was used to assess normality of data distribution. Paired comparisons of continuous variables were performed using the Wilcoxon signed-rank test. Correlations between RV pressure measurement methods were evaluated using Pearson’s correlation coefficient, and agreement was assessed via Bland–Altman analysis. A *p*-value < 0.05 was considered statistically significant. All statistical analyses were performed using IBM SPSS Statistics, version 29.

### 2.7. Ethical Statement

This study was approved by the Regional Scientific and Ethical Committee at the University of Debrecen, Clinical Center, and by the National Scientific and Ethical Committee of Hungary (Approval No: RKEB/IKEB: 6153-2022). All patients provided written informed consent prior to participation, in accordance with the Declaration of Helsinki.

## 3. Results

### 3.1. Baseline Characteristics of the Study Population

The study cohort consisted of 17 patients diagnosed with CTEPH. Baseline characteristics are shown in Table 1. The average age at the time of diagnosis was 61.4 years (range: 21–81), reflecting the disease’s prevalence across a broad adult age spectrum. Both sexes were represented (10 males and 7 females), and all patients were of white (Caucasian) ethnicity. Baseline body mass index (BMI) averaged 28.4 ± 5.0 kg/m^2^, ranging from normal weight to obesity, with several individuals exceeding a BMI of 35, highlighting the need to consider metabolic and cardiovascular comorbidities in clinical management.

At enrollment, the majority of patients were classified in NYHA functional class II–III, indicating moderate functional impairment, while some presented in class IV. The mean 6MWT distance was 330.6 ± 152.2 m, ranging from 42 to 548 m, underscoring significant variability in exercise tolerance. Baseline NT-proBNP levels were highly variable (mean: 4820.97 ± 10,288.15 pg/mL), consistent with different degrees of RV strain. Systolic pulmonary artery pressures measured by echocardiography, during BPA, and right heart catheterization averaged 72.1 ± 22.3 mmHg, 88.1 ± 11.9 mmHg, and 77.7 ± 23.5 mmHg, respectively.

Hemodynamic parameters obtained via right heart catheterization revealed elevated pulmonary vascular resistance (mean PVR: 675.4 ± 369.6 dyn·s·cm^−5^) and elevated mean pulmonary artery pressure (mPAP: 43.2 ± 8.6 mmHg), while pulmonary capillary wedge pressure (PCWP) and right atrial pressure (RAP) averaged 10.7 ± 3.4 mmHg and 7.9 ± 4.1 mmHg, respectively. Cardiac output (CO) and cardiac index (CI) were moderately reduced (mean CO: 4.44 ± 1.55 L/min; CI: 2.38 ± 0.79 L/min/m^2^), consistent with compromised RV performance.

Echocardiographic assessment (*n* = 97 studies) showed a mean TAPSE of 19.5 ± 5.1 mm, with several patients demonstrating values < 16 mm, indicating impaired RV systolic function. Basal RV diameter (RVD1) averaged 41.7 ± 6.1 mm, and right atrial area (RAA) was 25.2 ± 7.9 mm^2^, indicating RV dilation and pressure overload in many cases. The severity of tricuspid insufficiency (TI) ranged between grade I and III–IV.

**Figure 1:**
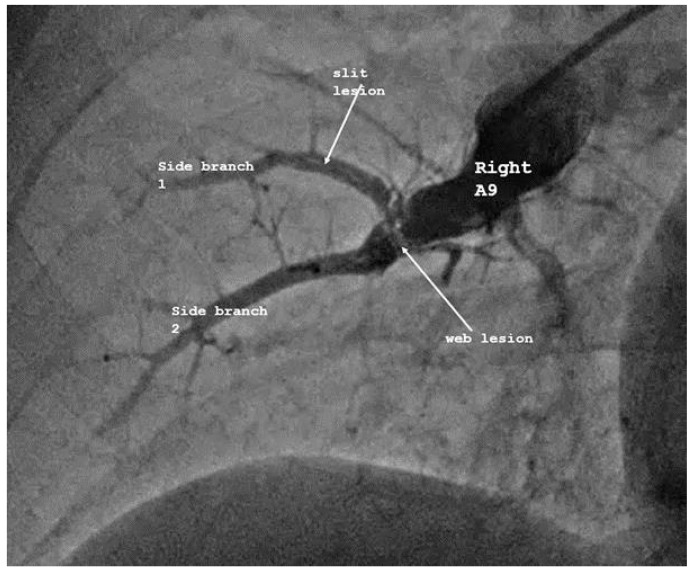
**Representative pulmonary angiographic image in CTEPH.** This pulmonary angiographic image of the right A9 segment artery and subsegmental branches illustrates the morphological characteristics of vascular lesions in CTEPH. The image highlights segmental or subsegmental filling defects, vascular cut-offs, and perfusion irregularities consistent with thromboembolic involvement. Slit- and web-type lesions are indicated by arrows. Lesion localization, extent, and contrast dynamics are shown to support diagnostic evaluation and therapeutic decision making in pulmonary hypertension or thromboembolic disease.

**Figure 2:**
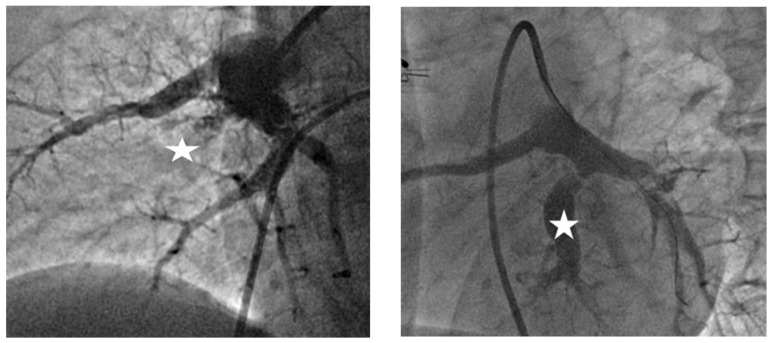
**Balloon pulmonary angioplasty (BPA).** In the left panel, the star shows the occluded right A9 segment. In the right panel, the guidewire is advanced into segment A8, following successful recanalization of A9, marked with the star.

Each CTEPH patient underwent an average of 3.13 ± 2.17 BPA sessions, with a mean of 12.0 ± 9.2 segmental dilatations and approximately 63% of affected segments treated. The follow-up of the whole study population is shown in Table 2 and Table 3. Representative angiographic images of morphological characteristics of vascular lesions in CTEPH are shown in Figure 1. Recanalization of an occluded pulmonary artery by BPA is demonstrated in Figure 2.

Medical history data revealed a high burden of thromboembolic disease. Six patients had a documented history of pulmonary embolism, while two were found to have inherited thrombophilia (prothrombin gene polymorphism 20210G/A heterozygous and heterozygous Factor V Leiden mutation). No patients had a history of splenectomy. ECG findings commonly showed right bundle branch block (RBBB) and RV strain patterns, with atrial fibrillation noted in a minority of cases.

The mean follow-up duration was 29.2 ± 10.2 months. During this period, six patients died, three due to non-cardiovascular causes including COVID-19, pneumonia, and septic shock.

### 3.2. Pulmonary Angioplasty and Therapeutic Response

As seen in Table 1, the number of BPA procedures per patient varied (up to 11 sessions). A total of 50 BPA procedures were performed in 17 patients diagnosed with CTEPH, with an average of 3.13 intervention sessions per patient. A total of 195 segmental/subsegmental dilatations were performed, with a mean of 12 dilatations per patient (SD = 9.16). The proportion of treated vs. affected segments was high (e.g., >75% in some patients), indicating extensive procedural effort and vascular involvement (Table 3).

PCWP values were collected and analyzed across the study cohort. The mean PCWP was 10.71 mmHg, with a standard deviation of 3.35 mmHg, indicating moderate variability within the patient population. The range of PCWP values was between 8 and 20 mmHg (Table 3). These measurements are consistent with expected hemodynamic profiles in patients with CTEPH, supporting the diagnosis of pre-capillary pulmonary hypertension in the majority of cases.

Patients were stratified into two groups based on their clinical response to BPA (Table 2). The well-responding group (*n* = 10) was defined as patients exhibiting an improvement of at least one NYHA class, and the poor-responding group (*n* = 7) included patients with minimal or no improvement in NYHA class.

Our analysis revealed notable differences between well- and poor-responding BPA patients. In the well-responding group, the 6MWT distance improved significantly, with an average increase of 60 ± 18 m compared to a marginal increase of 12 ± 10 m in the poor-responding group (*p* < 0.01). NT-proBNP levels decreased by an average of 40% in well-responders, whereas poor-responders showed only a 10% reduction (*p* < 0.01). Echocardiographic measurements demonstrated a significant improvement in RV function among well-responders; TAPSE increased from 16.0 ± 2.0 mm at baseline to 19.5 ± 2.5 mm at 6 months (*p* < 0.01), while the RV basal diameter decreased by 15% on average (*p* < 0.05). Conversely, poorly responding patients exhibited no significant change in TAPSE (from 16.5 ± 2.3 mm to 16.0 ± 2.5 mm, *p* > 0.05) and a trend toward increased RV dimensions.

**Figure 3:**
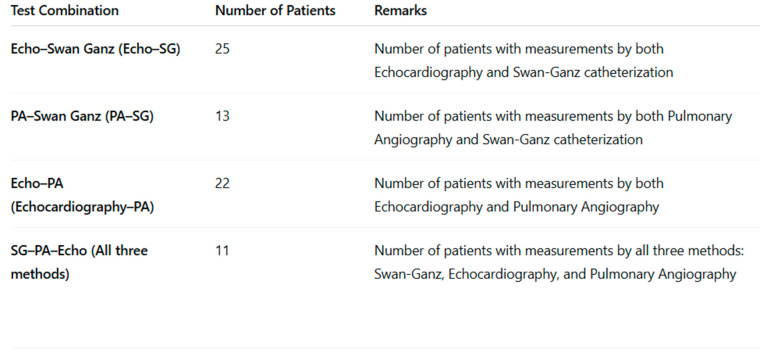
Simultaneous multimodal assessment of the right ventricular pressure in patient subgroups—the number of patients evaluated by multiple methods during the same hospitalization.

**Figure 4:**
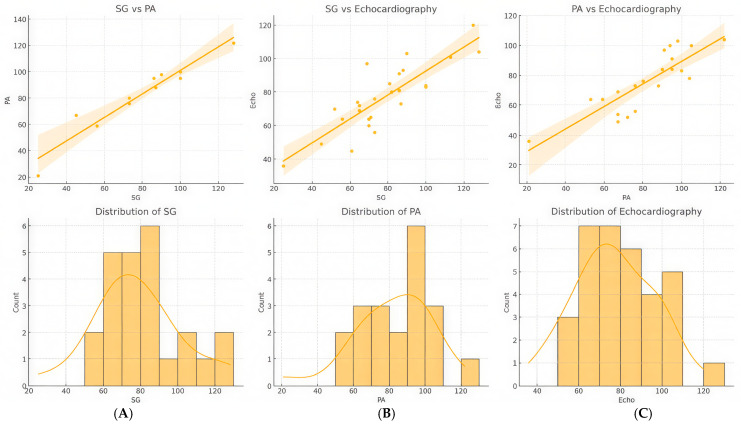
Simultaneous multimodal assessment of the right ventricular pressure in patient subgroups. Comprehensive comparison of pressure values obtained using three different methodologies: Swan-Ganz catheterization (SG), pulmonary artery pressure measurement during pulmonary angiography (PA), and echocardiographic estimation (Echo). Pressure values are given in mmHg. The top row displays scatter plots with regression lines to assess the correlation between the methods. (**A**) Measurements by both SG and PA (*n* = 13), (**B**) measurements by both SG and Echo (*n* = 25), (**C**) measurements by both PA and Echo (*n* = 22). The bottom row shows histograms with kernel density estimation (KDE) curves to illustrate the distribution patterns of each method’s measurements.

In the analysis of clinical data, three measurement techniques for assessing pulmonary artery pressure, Swan-Ganz (SG), pulmonary angiography (PA), and echocardiography, were compared (Table 3, Figure 3 and Figure 4). During the study period, a total of 97 echocardiographic examinations, 50 balloon pulmonary angioplasties (BPAs), and 43 Swan-Ganz catheterizations were recorded. Within the 48 h time frame, all three modalities—echocardiography, Swan-Ganz catheterization, and pulmonary angiography—were available for pressure comparison in 11 cases. Echocardiographic and Swan-Ganz-derived pressures were available for comparison in 25 cases, while pulmonary angiography and echocardiographic pressure estimations were performed within 48 h in 22 cases. In 13 cases, both Swan-Ganz catheterization and pulmonary angiography-derived pressure measurements were available within the defined time window.

The first scatter plot demonstrates a strong positive correlation between SG and PA readings, with a steep regression line and a narrow confidence interval, indicating high agreement between the two methods. This suggests that PA measurements can reliably reflect SG values in clinical settings. The second scatter plot, which compares SG and echocardiography, also shows a positive correlation, although the data points are more dispersed around the regression line. This wider spread suggests that, while echocardiography provides a reasonable estimate of SG values, there is greater variability, which is consistent with the known challenges of echocardiographic pressure estimation, such as operator dependency and technical limitations. Similarly, the third scatter plot, comparing PA and echocardiography, reveals a positive linear relationship but with slightly more dispersion compared to SG and echocardiography. This indicates moderate agreement between PA and echocardiographic measurements, further confirming the usefulness of echocardiography as a non-invasive method, though it may have slightly lower predictive precision than the direct catheter-based approach.

Histograms with kernel density estimation (KDE) curves were also used to assess the distribution of each measurement method. The histogram for SG values shows a relatively normal distribution, with most values concentrated around 70–90 mmHg. The KDE curve supports this, displaying a unimodal distribution with slight right skewness, suggesting a clinical population with varied pressure levels but a central tendency. In contrast, the PA distribution is broader and more evenly spread, as indicated by the KDE curve, reflecting a wider range of values and potential variability. The echocardiographic distribution, meanwhile, exhibits greater variability and multiple peaks, indicating the subjective nature of echocardiographic measurements and higher inter-observer variability. The flatter KDE curve for echocardiography further supports this notion.

Overall, the combination of scatter plots and histograms provides a robust assessment of the comparative behavior of the three measurement techniques. Swan-Ganz remains the reference standard due to its invasive precision, while PA and echocardiography offer useful approximations with varying levels of agreement. The scatter plots underscore correlation strength, while the histograms reveal potential variability in clinical application.

### 3.3. Correlation Analysis Between Right Heart Pressure Measurements Using Echocardiography, Swan-Ganz Catheterization, and Pulmonary Angiography

**Figure 5:**
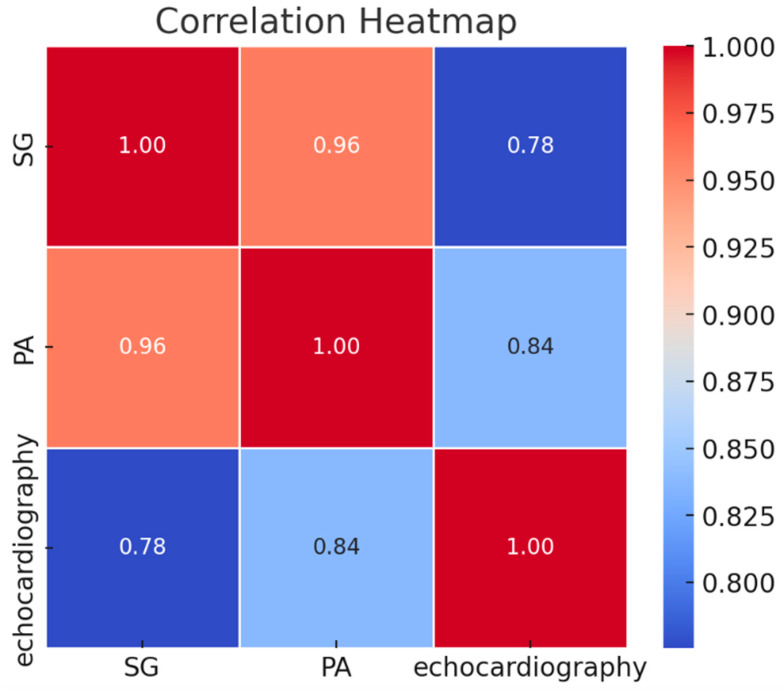
Correlation heatmap. Correlation between right heart pressures measured by different methods—shows strong positive relationships between SG, PA, and echocardiography.

Based on the collected data, statistical associations between right heart pressure values obtained via different diagnostic modalities were analyzed and visualized in a correlation matrix (heatmap format, Figure 5). The matrix includes three techniques: Swan-Ganz catheterization (SG)—invasive gold standard, pulmonary angiography (PA)—invasive pressure estimation, and transthoracic echocardiography (TTE)—non-invasive estimation method.

The correlation coefficients (Pearson’s r) quantify the linear relationship between each pair of methods. The results demonstrate varying degrees of correlation, with the strongest association observed between the two invasive techniques (SG and PA), and slightly weaker—but still significant—correlations involving echocardiographic estimates.

There is a very strong correlation between Swan-Ganz catheterization and pulmonary angiography-derived pressures (*r* = 0.96), indicating high consistency between these two invasive measurement techniques. The correlation between echocardiography and PA is slightly lower (*r* = 0.84) but still demonstrates a strong positive relationship. The weakest correlation is observed between echocardiographic and Swan-Ganz measurements (*r* = 0.78), which is still statistically strong but reflects the inherent limitations and variability of non-invasive estimation techniques.

Subgroup analysis revealed that, in patients with optimal acoustic windows, the correlation was even stronger (*r* = 0.88, *p* < 0.001). However, in patients with poor echocardiographic windows or irregular heart rhythms, discrepancies up to 10 mmHg were observed. In these cases, invasive measurements provided more consistent and reproducible data. Additionally, echocardiographic estimation tended to slightly underestimate RV pressure in patients with severe RV dilation.

## 4. Discussion

In CTEPH, accurate assessment of right heart pressures is crucial for diagnosis, risk stratification, and therapeutic decision making. Various modalities, including echocardiography, direct measurement during pulmonary angiography, and right heart catheterization, are employed to evaluate these pressures. Echocardiography serves as a non-invasive tool to estimate pulmonary artery pressures and assess RV function. However, right heart catheterization was considered the gold standard method for hemodynamic evaluation in pulmonary hypertension, providing definitive measurements of mPAP, PCWP, PVR, and CO. These parameters are essential for confirming the diagnosis of CTEPH and distinguishing it from other forms of pulmonary hypertension. Right ventricular pressure measurement plays a central role in the clinical management of patients with CTEPH. Elevated RV pressure indicates increased resistance in the pulmonary arteries, which is a hallmark of the disease, and helps assess the severity of pulmonary hypertension. It also provides insight into right heart function, as chronic pressure overload can lead to right ventricular dysfunction or failure. This parameter supports therapeutic decision making by guiding the selection of appropriate interventions, such as PEA, BPA, or targeted medical therapy. Moreover, serial RV pressure measurements allow clinicians to monitor disease progression and evaluate response to treatment over time. Importantly, persistently elevated RV pressures are associated with poorer prognosis, making this parameter valuable not only for diagnosis and follow-up but also for risk stratification.

Our findings indicate that echocardiographic estimation of RV pressure shows strong correlation with invasive measurements obtained by Swan-Ganz catheterization and PA in our CTEPH patient cohort. This supports the growing body of evidence that non-invasive methods, when performed under standardized conditions, can reliably monitor hemodynamic status in CTEPH. The overall mean differences between the modalities were clinically acceptable, suggesting that echocardiography may be a viable alternative for routine follow-up, reducing the need for invasive procedures.

There is a strong statistical correlation between right heart pressure measurements obtained via different modalities. Swan-Ganz catheterization and PA show a very high degree of agreement (*r* = 0.96), indicating consistency between these invasive techniques. Echocardiographic estimates correlate well with PA (*r* = 0.84), while the weakest—though still significant—correlation is observed between echocardiography and Swan-Ganz measurements (*r* = 0.78). The estimated right heart pressure derived from echocardiography is an indirect measurement and therefore less accurate, being operator-dependent, among other factors. Similarly, Swan-Ganz catheterization may also be prone to inaccuracies—the position of the catheter tip is not confirmed under fluoroscopy but inferred from the pressure waveform, and the overall setup is more complex, which may contribute to this variability. Nevertheless, no significant discrepancy between the echocardiographic and the two invasive measurements was found. These results confirm the reliability of invasive methods for precise hemodynamic assessment and support the use of echocardiography as a practical non-invasive tool, with the caveat that its estimates should be interpreted cautiously in cases requiring exact pressure values.

These data reflect the complexity and severity of CTEPH, emphasizing the need for individualized treatment strategies and close longitudinal monitoring.

**Clinical Interpretation:**

Our results underscore the reliability of invasive hemodynamic assessment (Swan-Ganz and PA) in evaluating pulmonary and right heart pressures while also supporting the utility of echocardiography as a non-invasive screening and follow-up tool. The slightly reduced correlation between echo and invasive methods highlights the importance of cautious interpretation of echocardiographic estimates, particularly when precise pressure values are critical for diagnosis or management (e.g., in suspected pulmonary hypertension).

In patients with suboptimal imaging conditions or significant RV remodeling, echocardiography may underestimate RV pressure, leading to potential misclassification of disease severity. This underscores the importance of individualizing the choice of monitoring modality based on patient-specific factors. Moreover, the integration of multiple measurement techniques may provide a more comprehensive assessment, particularly in borderline cases.

Our study also highlights the importance of using standardized protocols for echocardiographic measurements to minimize variability. Future research should focus on refining these protocols and exploring advanced imaging techniques, such as three-dimensional echocardiography (3D echo) or strain imaging, to further enhance the accuracy of non-invasive RV pressure and function estimation.

BPA has emerged as a vital therapeutic option for patients with inoperable CTEPH. However, clinical response to BPA varies considerably among patients. Identifying patient subgroups that may respond favorably to BPA is important. Patients with distal pulmonary artery obstructions, as opposed to proximal lesions, are more likely to benefit from BPA. Accurate hemodynamic assessment helps in selecting appropriate candidates for BPA, thereby optimizing clinical outcomes. In this prospective study we aimed to compare clinical, echocardiographic parameters, and NT-proBNP values between CTEPH patients who responded well to BPA and those who exhibited a less favorable response. Patients undergoing BPA were stratified into well-responding and poor-responding groups based on improvements in the NYHA functional class. Our extended analysis shows that well-responding patients demonstrated significant improvements in RV function (with increases in tricuspid annular plane systolic excursion [TAPSE] and reductions in RV diameters, RAA), along with marked reductions in NT-proBNP levels and improvements in 6MWT performance. These significant improvements observed in well-responders highlight the efficacy of BPA in patients with a favorable baseline hemodynamic profile. In contrast, poor-responding patients showed minimal or no improvements, with persistently elevated NT-proBNP, progressive RV dilation, and negligible change in functional capacity—this result likely reflects an advanced degree of RV remodeling and microvascular dysfunction. These observations suggest that baseline hemodynamic and laboratory biomarkers can help predict BPA efficacy, guiding patient selection and management strategies.

Furthermore, baseline NT-proBNP levels and RV dimensions were strong predictors of BPA response. Correlation analysis showed that higher baseline NT-proBNP levels correlated with a poorer increase in 6MWT distance (*r* = −0.68, *p* < 0.01) and slighter improvements in TAPSE (*r* = −0.63, *p* < 0.01). Additionally, the number of BPA sessions required was significantly higher in patients with elevated pulmonary arterial pressures at baseline (*p* < 0.05).

Our findings are consistent with the previous literature, which has identified NT-proBNP and echocardiographic indices as reliable predictors of outcome in CTEPH.

The strong inverse correlation between baseline NT-proBNP levels and functional improvement suggests that patients with a higher burden of cardiac stress are less likely to benefit from BPA. Moreover, the requirement for additional BPA sessions in patients with higher baseline pulmonary pressures supports the notion that a greater thrombotic burden may necessitate more intensive intervention.

Additionally, our data suggest that integrating baseline clinical parameters and NT-proBNP values can enhance patient stratification. For instance, combining NT-proBNP levels, RV dimensions, and coagulation profiles into a composite score may offer a more robust prediction model for BPA outcomes. This approach could facilitate early identification of patients at risk of poor response, prompting the consideration of adjunctive therapies or alternative interventions.

The mechanisms underlying the differential response to BPA are likely multifactorial, involving not only the extent of mechanical obstruction but also the degree of underlying microvascular remodeling and myocardial adaptability. Future research should focus on larger, multi-center studies to validate these findings and explore the potential of incorporating novel biomarkers into risk stratification models. In addition, the development of adjunctive pharmacological strategies targeting coagulation and fibrinolysis may improve outcomes in patients who are less responsive to BPA alone.

Ultimately, an integrated approach that combines clinical evaluation, echocardiography, and selective invasive measurements could optimize patient management, guiding timely therapeutic interventions and improving outcomes in CTEPH. Additional large-scale studies are warranted to validate our findings and to develop predictive models that incorporate both non-invasive and invasive data.

**Limitations of the Different Pressure Measurement Methods**

Echocardiographic estimation of right heart pressures has several important limitations. Accurate assessment depends heavily on the presence and quality of the tricuspid regurgitation (TR) jet; in cases where the TR signal is absent or poorly visualized, reliable pressure estimation becomes difficult or impossible. Right atrial pressure (RAP) is typically inferred based on inferior vena cava (IVC) diameter and collapsibility, which can be influenced by the patient’s volume status, respiratory effort, or intra-abdominal pressure, thereby introducing variability. Beat-to-beat variability due to arrhythmias or respiration can also impact the accuracy of Doppler-based assessments. Furthermore, echocardiography may be less accurate at higher pressure ranges and tends to either under- or overestimate pressures in patients with significant pulmonary hypertension. The presence of additional cardiac abnormalities, such as right ventricular dysfunction, pericardial disease, or structural anomalies, may further confound non-invasive pressure estimates. All echocardiographic studies in our cohort were performed using the same imaging platform (Philips Epiq 5) by a consistently trained team working within a structured echocardiography training program. Most studies were archived and re-analyzed using the vendor-independent TomTec software (built number 493962), which enables automated and reproducible measurements. This integrated approach—including team-based image review, consistent methodology, and advanced post-processing—significantly reduced both inter- and intraobserver variability, thereby improving the reliability of serial assessments in our CTEPH population.

Despite being considered the gold standard for hemodynamic assessment, Swan-Ganz catheterization has inherent limitations and may be associated with complications. As an invasive procedure, it carries risks including bleeding, infection, arrhythmias, pulmonary artery rupture, and thromboembolism. Accurate pressure measurement depends on correct catheter positioning and waveform interpretation, both of which require substantial operator expertise. Technical issues such as overwedging or catheter migration can result in inaccurate readings. Moreover, hemodynamic data represent only a single time point and may be influenced by sedation, mechanical ventilation, or acute hemodynamic fluctuations, potentially limiting their applicability to a patient’s baseline status. While Swan-Ganz catheterization enables direct measurement of pulmonary artery pressures and pulmonary capillary wedge pressure (PCWP), interpretation can be challenging in specific clinical contexts such as mitral valve disease, pulmonary veno-occlusive disease, or complex congenital heart disease. Thermodilution-based cardiac output measurement may be unreliable in cases of severe hypothermia, significant tricuspid regurgitation, right-to-left intracardiac shunt, or when pulmonary blood flow is altered by acute respiratory distress syndrome (ARDS) or pulmonary edema. Interpretation requires considerable experience, and the risk of misinterpretation should be recognized.

Pressure measurements obtained during pulmonary angiography are typically performed using diagnostic catheters that are not specifically designed for accurate hemodynamic monitoring. These catheters often lack the sensitivity and calibration precision of dedicated devices such as Swan-Ganz catheters, which may limit the reliable detection of subtle changes in pulmonary artery pressures. Furthermore, angiography-based pressure assessments are generally confined to the main or proximal pulmonary arteries and may not adequately reflect the hemodynamic impact of more distal, segmental, or subsegmental vascular obstruction—a limitation particularly relevant in patients with predominantly peripheral-type CTEPH. These isolated pressure readings obtained by pulmonary angiography also do not give the possibility of calculating further parameters such as pulmonary vascular resistance (PVR) or cardiac output, which are necessary for comprehensive hemodynamic evaluation. In addition, several procedural factors—including catheter positioning, contrast administration, and the patient’s intraprocedural hemodynamic status—can influence the accuracy and reproducibility of pressure recordings during angiography.

## 5. Conclusions

Echocardiographic estimation of right ventricular pressure correlates well with invasive measurements in CTEPH patients and can be reliably used for routine follow-up when standardized protocols are adhered to.

Our study identified distinct clinical, echocardiographic, and biochemical differences between patients who responded well to balloon pulmonary angioplasty (BPA) and those who showed limited improvement. Well-responding patients experienced significant gains in functional capacity, reflected by a marked increase in a 6 min walk distance and a substantial reduction in NT-proBNP levels. Echocardiographic evaluation also revealed improved right ventricular (RV) function, as evidenced by increased TAPSE and reduced RV dimensions. In contrast, poor responders exhibited persistently elevated NT-proBNP levels, minimal functional gains, and no significant improvement in RV parameters, likely indicating advanced myocardial remodeling and microvascular dysfunction.

## Figures and Tables

**Table 1:** Patients’ baseline characteristics.

Parameter	Mean ± SD	Range
BMI	28.39 ± 5.00	20.86–37.11
Systolic BP (mmHg)	125.56 ± 17.39	104–153
Age at Diagnosis (years)	61.41 ± 14.34	21–81
NYHA Class	2.76 ± 0.83	1–4
6MWT Distance	330.63 ± 152.17	42.0–548.0
NT-proBNP (pg/mL)	4820.97 ± 10,288.15	82.0–43,722.0
PAPs (mmHg)-echo	72.07 ± 22.25	43.0–128.0
PAPs (mmHg)-BPA	88.10 ± 11.90	76.0–104.0
PAPs (mmHg)-SG	77.69 ± 23.49	41.0–111.0
PVR ^1^ (dyn·s·cm^−5^)	675.38 ± 369.60	272.0–1786.0
PVRI ^1^ (dyn·s·cm^−5^·m^2^)	1275.19 ± 691.34	518.0–3232.0
PCWP ^1^ (mmHg)	10.71± 3.35	8–20
Mean Pulmonary Artery Pressure ^1^	43.23 ± 8.56	24.0–80.0
Right Atrial Pressure ^1^	7.88 ± 4.13	2.0–21.0
Cardiac Output ^1^ (CO, L/min)	4.44 ± 1.55	1.2–6.9
Cardiac Index ^1^ (CI, L/min/m^2^)	2.38 ± 0.79	0.7–4.6
Number of BPA Procedures	3.13 ± 2.17	1–11
Total Number of Dilatations	12.00 ± 9.16	3–44
% of Treated Segments	62.96 ± 10.84	49.90–78.12
TAPSE (mm)	19.50 ± 5.15	10.0–26.0
RVD1 (mm)	41.75 ± 6.08	39.0–52.0
RAA (mm^2^)	25.16 ± 7.89	15.0–36.5
TI Severity	II–III	I–III–IV
Left Atrium Diameter	36.25 ± 5.53	30.0–45.0

^1^ Values are obtained via right heart catheterization using a Swan-Ganz catheter. BMI: body mass index; BP: blood pressure; NYHA: New York Heart Association; 6MWT: 6 min walk test; PAP: systolic pulmonary arterial pressure; PVR (I): pulmonary vascular resistance (index); PCWP: pulmonary capillary wedge pressure; BPA: balloon pulmonary angioplasty; TAPSE: tricuspid annular plane systolic excursion; RVD1: right ventricular diameter 1; RAA: right atrial area; TI: tricuspid regurgitation; and N: 17 patients’ data.

**Table 2:** Differences between the well-responding and poor-responding groups.

	Well-Responding Group (*n* = 10)	Poor-Responding Group (*n* = 7)	*p*
BMI	27.39 ± 5.35	29.81 ± 4.46	0.364
Systolic BP (mmHg)	128.00 ± 19.33	121.50 ± 14.25	0.428
Age at Diagnosis (years)	60.30 ± 16.70	63.00 ± 11.162	0.740
NYHA Class	2.70 ± 0.82	2.86 ± 0.89	0.887
6MWT Distance	317.80 ± 176.58	352.00 ± 111.64	0.792
NT-proBNP (pg/mL)	5077.78 ± 10,569.13	2598 ± 3217.74	0.740
PAPs (mmHg)-echo	80.00 ± 22.63	62.43 ± 15.96	0.114
PAPs (mmHg)-BPA	89.88 ± 10.40	81 ± 19.80	0.533
PAPs (mmHg)-SG	88.33 ± 23.02	64 ± 16.85	0.042
PVR (dyn·s·cm^−5^) ^2^	793.56 ± 435.25	523.43 ± 172.5	0.210
PVRI (dyn·s·cm^−5^·m^2^) ^2^	1442.67 ± 805.03	1059.86 ± 484.38	0.408
PCWP (mmHg) ^2^	12.00 ± 3.50	8.86 ± 2.19	0.681
Mean Pulmonary Artery Pressure ^2^	44.57 ± 10.23	41.67 ± 6.68	0.267
Cardiac Output (CO, L/min) ^2^	4.21 ± 1.44	4.73 ± 1.73	0.351
Cardiac Index (CI, L/min/m^2^)	2.31 ± 0.77	2.47 ± 0.88	0.606
Number of BPA Procedures	3.56 ± 2.96	2.37 ± 1.19	0.042
Total Number of Dilatations	14.60 ± 10.35	8.29 ± 5.13	0.193
% of Treated Segments	69.24 ± 19.14	45.47 ± 30.15	0.161
TAPSE (mm)	18.33 ± 3.84	21.00 ± 6.48	0.470
RVD1 (mm)	45.00 ± 3.08	37.57 ± 6.13	0.023
RAA (mm^2^)	26.88 ± 6.54	22.96 ± 9.40	0.299
TI Severity	II–III	II–III	
Left Atrium Diameter	36.89 ± 4.43	36.71 ± 7.08	0.873

Statistical analysis: Mann–Whitney test. *p*-value <0.05 is considered statistically significant. ^2^ Values are obtained via right heart catheterization. BMI: body mass index; BP: blood pressure; NYHA: New York Heart Association; 6MWT: 6 min walk test; PAP: systolic pulmonary arterial pressure; PVR (I): pulmonary vascular resistance (index); PCWP: pulmonary capillary wedge pressure; BPA: balloon pulmonary angioplasty; TAPSE: tricuspid annular plane systolic excursion; RVD1: right ventricular diameter 1; RAA: right atrial area; TI: tricuspid regurgitation; and *n* = 17 patient.

**Table 3:** Clinical, echocardiographic, hemodynamic, and BPA-assessed parameters during follow-up.

Parameter		Baseline (*n* = 17)	1-Year Follow-up (*n* = 17)	2-Year Follow-up (*n* = 15)	3-Year Follow-up (*n* = 8)
NYHA ^1^		2.76 ± 0.83	2.69 ± 0.87	2.57 ± 0.94	2.86 ± 1.07
Clinical improvement ^2^			0.63 ± 0.72	0.79 ± 0.70	0.71 ± 0.49
6MWT (m)		330.63 ± 152.17	350.33 ± 155.73	323.92 ± 138.46	309.00 ± 174.48
NT-proBNP (pg/mL)		4820.97 ± 10,288.15	2932.30 ± 3388.65	2177.07 ± 2100.31	1798.29 ± 1953.28
**SG**	PAPs (mmHg)	88.10 ± 11.90	72.60 ± 20.38	73.31 ± 22.66	76.17 ± 30.76
	PAPd (mmHg)	26.25 ± 9.26	26.40 ± 8.49	27.62 ± 9.47	29.86 ± 12.76
	PAPm (mmHg)	43.23 ± 8.55	41.70 ± 8.65	41.38 ± 9.41	43.00 ± 11.79
	PCWP (mmHg)	10.71 ± 3.35	10.93 ± 2.40	11.23 ± 2.45	10.71 ± 3.50
	PVR (dyn·s·cm^−5^)	675.38 ± 369.60	648.93 ± 374.91	639.00 ± 320.66	684.86 ± 503.41
	PVRI (dyn·s·cm^−5^·m^2^)	1275.19 ± 691.34	1254.07 ± 711.95	1204.46 ± 544.86	1293.14 ± 941.22
	CO (L/min)	4.44 ± 1.55	4.50 ± 1.35	4.78 ± 1.65	4.71 ± 1.35
	CI (L/min/m^2^)	2.38 ± 0.79	2.41 ± 1.15	2.41 ± 9.16	2.55 ± 6.49
**PA-BPA**	Number of involved segments	10.88 ± 5.67			
	Proportion of dilated segments relative to affected segments (%)		43.79 ± 24.27	60.78 ± 19.60	63.31 ± 19.36
	RVP syst. (mmHg)	89.00 ± 13.47	85.73 ± 12.36	78.30 ± 12.23	84.17 ± 12.00
	RVP diast. (mmHg)	31.56 ± 9.38	25.17 ± 7.69	25.40 ± 7.11	25.33 ± 8.19
	RVP mean (mmHg)	52.56 ± 7.69	46.58 ± 7.51	45.50 ± 7.26	47.50 ± 8.16
**Echocardiography**	RVP (mmHg)	77.33 ± 7.69	77.73 ± 8.99	77.85 ± 7.23	81.00 ± 7.55
	TAPSE (mm)	19.50 ± 5.15	20.00 ± 4.61	18.54 ± 4.79	20.43 ± 5.25
	RVD1 (mm)	41.75 ± 6.08	41.40 ± 6.12	39.62 ± 4.41	37.14 ± 4.13
	RAA (cm^2^)	25.16 ± 5.53	23.16 ± 8.11	26.48 ± 12.14	22.66 ± 8.40

^1^ NYHA functional class. ^2^ Clinical improvement is assessed based on the patient’s subjective evaluation, comparing their current health status to their condition during the previous hospitalization. A score of 1 is assigned for perceived improvement and 0 for no change or deterioration. The table contains the data of 17 CTEPH patients during the follow-up period. Values are expressed as average ± SD, statistics: Wilcoxon test, *n* = 17. BMI: body mass index; BP: blood pressure; NYHA: New York Heart Association; 6MWT: 6 min walk test; PAP: systolic pulmonary arterial pressure; PVR (I): pulmonary vascular resistance (index); PCWP: pulmonary capillary wedge pressure; BPA: balloon pulmonary angioplasty; TAPSE: tricuspid annular plane systolic excursion; RVD1: right ventricular diameter 1; RAA: right atrial area; TI: tricuspid regurgitation; and N: 17 patients.

## Data Availability

The data that support the findings of this study are available upon request from the corresponding author. The data are not publicly available due to general data protection regulations.

## Abbreviations

The following abbreviations are used in this manuscript:

3D TTE	Three-Dimensional Transthoracic Echocardiography
BMI	Body Mass Index
BP	Blood Pressure
BPA	Balloon Pulmonary Angioplasty
CI	Cardiac Index
CO	Cardiac Output
CTEPH	Chronic Thromboembolic Pulmonary Hypertension
ESC	European Society of Cardiology
IQR	Interquartile Range
IVC	Inferior Vena Cava
mPAP	Mean Pulmonary Artery Pressure
NT-proBNP	N-terminal pro-B-type Natriuretic Peptide
NYHA	New York Heart Association
PA	Pulmonary Angiography
PAP	Pulmonary Artery Pressure
PEA	Pulmonary Endarterectomy
PCWP	Pulmonary Capillary Wedge Pressure
PVR	Pulmonary Vascular Resistance
PVRI	Pulmonary Vascular Resistance Index
RAP	Right Atrial Pressure
RVD1	Right Ventricular Diameter at Basal Level
RAA	Right Atrial Area
RV	Right Ventricular
SD	Standard Deviation
SG	Swan-Ganz Catheterization
sPAP	Systolic Pulmonary Artery Pressure
TAPSE	Tricuspid Annular Plane Systolic Excursion
TR	Tricuspid Regurgitation
TRV	Tricuspid Regurgitation Velocity

## References

[1] Delcroix M., Torbicki A., Gopalan D., Sitbon O., Klok F.A., Lang I., Jenkins D., Kim N.H., Humbert M., Jais X. (2021). ERS statement on chronic thromboembolic pulmonary hypertension. Eur. Respir. J..

[2] Teerapuncharoen K., Bag R. (2022). Chronic Thromboembolic Pulmonary Hypertension. Lung.

[3] Bazmpani M.A., Arvanitaki A., Toumpourleka M., Pitsiou G., Panagiotidou E., Mouratoglou S.A., Sianos G., Hadjimiltiades S., Pitsis A., Mayer E. (2018). Epidemiology and management of chronic thromboembolic pulmonary hypertension: Experience from two expert centers. Hell. J. Cardiol..

[4] Shahidi P., Mentzel L., Blazek S., Sulimov D., Thiele H., Fengler K. (2024). From Pulmonary Embolism to Chronic Thromboembolic Pulmonary Hypertension: A Pathophysiological Approach. Rev. Cardiovasc. Med..

[5] Matthews D.T., Hemnes A.R. (2016). Current concepts in the pathogenesis of chronic thromboembolic pulmonary hypertension. Pulm. Circ..

[6] Bonderman D., Wilkens H., Wakounig S., Schäfers H.-J., Jansa P., Lindner J., Simkova I., Martischnig A.M., Dudczak J., Sadushi R. (2009). Risk factors for chronic thromboembolic pulmonary hypertension. Eur. Respir. J..

[7] Ghani H., Pepke-Zaba J. (2024). Chronic Thromboembolic Pulmonary Hypertension: A Review of the Multifaceted Pathobiology. Biomedicines.

[8] Labrada L., Vaidy A., Vaidya A. (2023). Right ventricular assessment in pulmonary hypertension. Curr. Opin. Pulm. Med..

[9] Simonneau G., Torbicki A., Dorfmüller P., Kim N. (2017). The pathophysiology of chronic thromboembolic pulmonary hypertension. Eur. Respir. Rev..

[10] Correale M., Chirivì F., Bevere E.M.L., Tricarico L., D’Alto M., Badagliacca R., Brunetti N.D., Vizza C.D., Ghio S. (2024). Endothelial Function in Pulmonary Arterial Hypertension: From Bench to Bedside. J. Clin. Med..

[11] Simeone B., Maggio E., Schirone L., Rocco E., Sarto G., Spadafora L., Bernardi M., D’Ambrosio L., Forte M., Vecchio D. (2024). Chronic Thromboembolic Pulmonary Hypertension: The Diagnostic Assessment. Front. Cardiovasc. Med..

[12] Lang I.M., Madani M. (2014). Update on chronic thromboembolic pulmonary hypertension. Circulation.

[13] Ruaro B., Baratella E., Caforio G., Confalonieri P., Wade B., Marrocchio C., Geri P., Pozzan R., Andrisano A.G., Cova M.A. (2022). Chronic Thromboembolic Pulmonary Hypertension: An Update. Diagnostics.

[14] Grünig E., Peacock A.J. (2015). Imaging the heart in pulmonary hypertension: An update. Eur. Respir. Rev..

[15] Mayer E., Jenkins D., Lindner J., D’armini A., Kloek J., Meyns B., Ilkjaer L.B., Klepetko W., Delcroix M., Lang I. (2011). Surgical management and outcome of patients with chronic thromboembolic pulmonary hypertension: Results from an international prospective registry. J. Thorac. Cardiovasc. Surg..

[16] Ghofrani H.A., D’ARmini A.M., Grimminger F., Hoeper M.M., Jansa P., Kim N.H., Mayer E., Simonneau G., Wilkins M.R., Fritsch A. (2013). Riociguat for the treatment of chronic thromboembolic pulmonary hypertension. N. Engl. J. Med..

[17] Rudski L.G., Lai W.W., Afilalo J., Hua L., Handschumacher M.D., Chandrasekaran K., Solomon S.D., Louie E.K., Schiller N.B. (2010). Guidelines for the echocardiographic assessment of the right heart in adults: A report from the American Society of Echocardiography. J. Am. Soc. Echocardiogr..

[18] Humbert M., Kovacs G., Hoeper M.M., Badagliacca R., Berger R.M.F., Brida M., Carlsen J., Coats A.J.S., Escribano-Subias P., Ferrari P. (2022). 2022 ESC/ERS Guidelines for the diagnosis and treatment of pulmonary hypertension. Eur. Heart J..

[19] Mukherjee M., Rudski L.G., Addetia K., Afilalo J., D’aLto M., Freed B.H., Friend L.B., Gargani L., Grapsa J., Hassoun P.M. (2025). Guidelines for the Echocardiographic Assessment of the Right Heart in Adults and Special Considerations in Pulmonary Hypertension: Recommendations from the American Society of Echocardiography. J. Am. Soc. Echocardiogr..

[20] Seyyedi S.R., Mozafari M., Sharif-Kashani B., Sadr M., Emami H., Mehrazmay A. (2022). Correlation of Echocardiographic and Right Heart Catheterization Estimations of Pulmonary Artery Systolic Pressure. Tanaffos.

[21] Rich J.D., Shah S.J., Swamy R.S., Kamp A., Rich S. (2011). Inaccuracy of Doppler echocardiographic estimates of pulmonary artery pressures in patients with pulmonary hypertension: Implications for clinical practice. Chest.

[22] Madani M., Mayer E., Fadel E., Jenkins D.P. (2016). Pulmonary Endarterectomy. Patient Selection, Technical Challenges, and Outcomes. Ann. Am. Thorac. Soc..

[23] Saouti N., Morshuis W.J., Heijmen R.H., Snijder R.J. (2012). Long-term outcome after pulmonary endarterectomy for chronic thromboembolic pulmonary hypertension. J. Thorac. Cardiovasc. Surg..

[24] Faccioli E., Verzeletti V., Perazzolo Marra M., Boscolo A., Schiavon M., Navalesi P., Rea F., Dell’Amore A. (2022). Pulmonary Endarterectomy for Chronic Thromboembolic Pulmonary Hypertension: A Systematic Review of the Most Updated Literature. J. Clin. Med..

[25] Galié N., Humbert M., Vachiéry J.-L., Gibbs S., Lang I., Torbicki A., Simonneau G., Peacock A., Noordegraaf A.V., Beghetti M. (2015). 2015 ESC/ERS Guidelines for the diagnosis and treatment of pulmonary hypertension. Eur. Respir. J..

[26] Delcroix M., Lang I., Pepke-Zaba J., Jansa P., D’Armini A.M., Snijder R., Bresser P., Torbicki A., Mellemkjaer S., Lewczuk J. (2016). Long-term outcome of patients with chronic thromboembolic pulmonary hypertension: Results from an international prospective registry. Circulation.

[27] Kwon W., Yang J.H., Park T.K., Chang S.A., Jung D.S., Cho Y.S., Kim S.M., Kim T.J., Park H.Y., Choi S.H. (2018). Impact of Balloon Pulmonary Angioplasty on Hemodynamics and Clinical Outcomes in Patients with Chronic Thromboembolic Pulmonary Hypertension: The Initial Korean Experience. J. Korean Med. Sci..

[28] Suntharalingam J., Goldsmith K., Toshner M., Doughty N., Sheares K.K., Hughes R., Jenkins D., Pepke-Zaba J. (2007). Role of NT-proBNP and 6MWD in chronic thromboembolic pulmonary hypertension. Respir. Med..

